# Effects of coenzyme Q10 supplementation on myopathy in statin-treated patients: a systematic review and meta-analysis

**DOI:** 10.1017/jns.2025.10043

**Published:** 2025-10-10

**Authors:** Svenja Kovacic, Sandra D. Habicht, Gunter Peter Eckert

**Affiliations:** Department of Nutritional Science, Justus Liebig University Giessen, Giessen, Germany

**Keywords:** Coenzyme Q10, HMG-CoA reductase-inhibitors, Hypercholesterolemia, Myopathy, Statins, Statin-associated muscle symptoms, Ubiquinone, BPI, Brief pain inventory, CENTRAL, Cochrane central register of controlled trials, CK, Creatine kinase, CoQ10, Coenzyme Q10, Corr, Correlation coefficient, CVD, Cardiovascular diseases, L/P Ratio, lactate to pyruvate ratio, MeSH, Medical subject heading, n-3 FA, n-3-fatty acids, PIS, Pain interference score, PSS, Pain severity score, RCTs, randomised controlled trials, ROS, reactive oxygen species, SAMS, statin-associated muscle symptoms, U.S., United states, VAS, Visual analogue scale, WMD, Weighted mean difference

## Abstract

Statins are effective drugs for lowering hypercholesterolemia and preventing cardiovascular diseases. They can cause various side effects, in particular statin-associated muscle symptoms (SAMS) associated with mitochondrial dysfunction and micronutrient depletion. The aim of this systematic review and meta-analysis was to investigate the efficacy of a supplementation with Coenzyme Q10 (CoQ10) against SAMS in statin-treated patients. A systematic literature search was performed in Medline and Cochrane Library in August 2024. Studies were selected for a meta-analysis according to the following criteria: randomised controlled trials (RCTs), adults taking statins (any type and dose), supplementation of CoQ10, a comparable control group, and muscle pain as outcome criterion. Cochrane Risk of Bias tool was used for bias assessment. Seven RCTs with 389 patients in total were included in this meta-analysis. The selected studies included 35 to 76 patients and had a duration ranging from 30 to 90 days with CoQ10 dosages ranging from 100 to 600 mg per day. Results show a significant reduction of SAMS in four trials and no significant change in three trials. Overall, a significant reduction in SAMS, measured as pain intensity, after CoQ10 supplementation was found: weighted mean difference (WMD) −0.96 (95% Confidence Interval −1.88; −0.03), *p* < 0.05. Supplementation of CoQ10 can reduce muscle pain in patients with SAMS, which is relevant for their well-being and treatment continuation. More research is needed for evidence-based recommendations.

## Introduction

As cardiovascular diseases (CVD) remain the leading cause of global mortality,^(1)^ treatment of CVD and a reduction of risk factors, including hypercholesterolemia and hyperglycaemia, are essential to reduce the individual and economic health burden.^(2)^ Besides nutritional interventions and strategies against physical inactivity and smoking, drug therapy is advised in the prevention of CVD.^(3,4)^ The first-line drugs to counteract hypercholesterolemia are statins, which are among the most prescribed medication in the United States (U.S.) with increasing prescription numbers, highlighting its relevance in population-wide risk reduction.^(5,5,6)^ However, in addition to their beneficial cholesterol-lowering effects, statins cause both pleiotropic and adverse side effects that are significantly linked to impairments of mitochondrial function.^(7)^

Adverse drug reactions of statins comprise, among others, increased reactive oxygen species (ROS) production,^(8)^ increased blood levels of liver transaminases^(8)^ and creatine kinase (CK),^(9)^ and the occurrence of statin-associated muscle symptoms (SAMS).^(9)^ SAMS are prevalent in 5–20 % of statin taking patients and cover a range of mild to moderate muscle symptoms, including muscle pain, muscle cramps, muscle weakness, and muscle stiffness.^(10)^ In severe cases, myopathy can lead to rhabdomyolysis with subsequent renal failure.^(11)^ Consequently, the development of SAMS interferes with the patient’s quality of life and can lead to reduction of statin dose or discontinuation of statin therapy.^(10,12)^

Several causative mechanisms are discussed for the development of SAMS,^(9)^ but a statin-induced reduction of Coenzyme Q10 (CoQ10) levels is of particular interest.^(13)^ Statins (3-Hydroxy-3-methylglutaryl CoA reductase inhibitors) inhibit the endogenous synthesis of cholesterol by inhibiting the rate-limiting enzyme of the mevalonate pathway.^(14)^ Thus, statins intervene very early in the mevalonate metabolic pathway and inhibit the formation of intermediary products including geranylgeranyl pyrophosphate. This isoprenoid is essential for the endogenous synthesis of CoQ10.^(15)^ As CoQ10 is only supplied in small quantities with food and is predominantly synthesised endogenously,^(16,17)^ statin intake can lead to reduced CoQ10 levels.^(17)^

CoQ10, also known as Ubiquinone, is a fat-soluble vitamin-like cofactor. In physiological conditions, CoQ10 has structural and antioxidant properties and plays a central role as an electron transporter in the respiratory chain of mitochondria.^(16)^ Decreased CoQ10 levels are considered to disturb the mitochondrial electron transport, resulting in a limited adenosine triphosphate (ATP) synthase activity and thus leading to an impaired energy metabolism and mitochondrial dysfunction.^(16,18,19)^ Mitochondrial dysfunction in muscle cells can lead to altered biochemical parameters such as a higher lactate to pyruvate ratio (L/P ratio), but also to development of myopathic symptoms.^(20)^ In order to improve the mitochondrial function and to reduce SAMS, the supplementation of CoQ10 in statin-treated patients has been discussed for several years.^(21)^ As selenium is involved in the regeneration of CoQ10 and supports antioxidant properties, a supplementation of selenium solely or in combination with CoQ10 in statin-treated patients has also been discussed in the literature.^(22,23)^ However, data on selenium supplementation and SAMS is still insufficient for further literature analyses.

Although CoQ10 supplementation seems to be a plausible strategy to prevent SAMS,^(13,19)^ results from randomised controlled trials (RCTs) and previous meta-analyses show contradictory effects of CoQ10 supplementation on SAMS.^(24–27)^ Thus, the main objective of this systematic review and meta-analysis is to summarise the most recent clinical trials focusing on the effect of an oral supplementation with CoQ10 in patients with SAMS on myopathic pain intensity as a relevant clinical outcome for patients. The results of the systematic literature research are presented in the form of an up-to-date meta-analysis.

## Methods

### Search strategy and study selection

This review and meta-analysis was conducted based on the PRISMA statement^(28)^ and has been registered on the PROSPERO register (registration number CRD42023467604).^(29)^ The literature search process started in June 2022 with a comprehensive search for RCTs that analyse the effects of CoQ10 in patients with statin treatment. A systematic search in Medline and Cochrane Central Register of Controlled Trials (CENTRAL) was performed using both free-hand search terms and Medical Subject Heading (MeSH) terms (**supplementary data 1**). The search was re-run in August 2024 to include all trials published by the end of July 2024 and was checked by all study investigators to minimise selection bias.

Studies have been selected in accordance with the following inclusion criteria:

- RCTs
- adults ≥ 18 years with statin intake
- supplementation of CoQ10 as intervention
- placebo-controlled, or similar study arm that differs from the intervention group only in the intake of CoQ10
- muscle pain intensity as outcome criteria measured by pain rating scores

Studies were excluded if the following criteria applied:

- control group differing in patient characteristics
- beginning of statin intake only at trial start
- incomplete reporting of methods and outcome measurements (not provided when authors could be successfully contacted)

**Figure 1.**
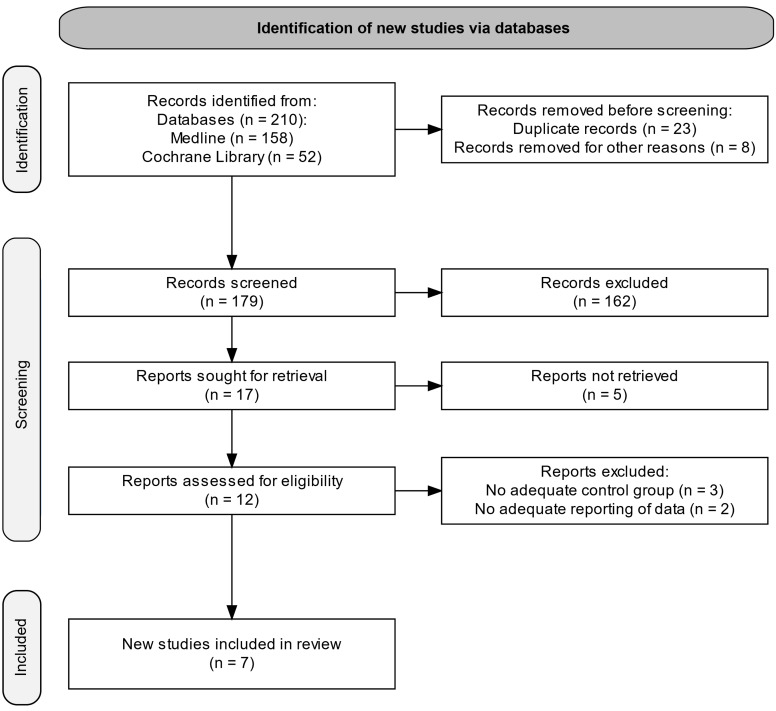
PRISMA Flow chart of search output and study selection process.

The output of the search process is presented in PRISMA Flow chart (Figure 1). A review protocol was not prepared.

### Data extraction process and quality assessment

Data extraction was performed by using Cochrane’s checklist of items to retrieve all information that is relevant for further analysis.^(30)^ The primary outcome of this analysis is the intensity of myopathic pain. Studies were included when muscle pain was measured before and after study period with scores such as the Visual Analogue Scale (VAS) or the Pain Severity Score (PSS). While the VAS results from pointing out the individual pain intensity on a measuring scale,^(31)^ the PSS is calculated from the Brief Pain Intensity questionnaire.^(32)^ Both scores rate the individual pain intensity on a scale from 0 (no pain) to 10 (worst pain) and have been validated in external analyses.^(31,32)^ One of the included studies by Tóth et al.^(33)^ measured muscle symptoms with a similar score scaling from 0 to 10 developed by the study team.^(33)^ When studies measured pain using the Brief Pain Inventory (BPI) questionnaire or its short form, only the results of the PSS were used for this analysis. The BPI consists of two parts; namely a PSS and a Pain Interference Score (PIS), which assesses the interference of pain with daily activities^(32)^ The results of the PIS were, however, not included in this analysis, because the PIS was reported only in two studies and the results were not comparable with the scores of the other studies.

Mitochondrial markers such as the lactate to pyruvate (L/P) ratio were considered as interesting secondary outcome criteria. However, trials measuring the L/P ratio did not meet the inclusion criteria of this review and data were reported inconsistently. Thus, the L/P ratio could not be included in the final analysis.

The data extraction process was performed by two investigators (SK and SH) to ensure the correctness of the extracted data. In case of more than two study arms, the respective study arms comparing CoQ10 supplementation to a control have been selected for this analysis. As there was relevant information not reported in the publication by Skarlovnik et al.,^(34)^ the authors have been contacted and asked for the provision of the required data. Potential bias in the included studies was assessed according to the following five domains: randomisation process, deviations from the intended interventions, missing outcome data, measurement of the outcome and selection of the reported results. In order to evaluate the potential bias on the individual study level and to summarise the results, the RoB2 online tool was used.^(35)^ Risk of bias assessment was conducted by SK and SH.

## Statistical analysis

Meta-analysis of changes in muscle pain intensity after CoQ10 supplementation compared to the control group was performed based on weighted mean difference (WMD) of continuous outcome data and 95 % Confidence Interval (CI) as effect size. Meta-analysis was conducted according to the inverse-variance approach, including both means and SD of changes from baseline.^(36)^ Data that was reported as SE was converted to SD according to the Cochrane handbook chapter 6.5.2.2.^(30)^ For most of the included studies, the SDs for the changes in myopathy scores from baseline have not been reported and were calculated with the aid of a correlation coefficient (Corr) according to the Cochrane Handbook chapter 6.5.2.3 and chapter 6.5.2.8.^(30)^ The Corr expresses the similarity between measurements pre- and post-intervention across participants.^(36)^ In this analysis, a Corr of 0.7 has been computed first and was used for further calculations of missing SDs. As in a former meta-analysis a Corr of 0.5 had been imputed,^(25)^ a sensitivity analysis with different values for Corr ranging from 0.5 to 0.9 was performed. By performing a leave-one-out sensitivity analysis where the meta-analysis was repeated seven times with one study not being included each time, the overall robustness to the study selection was tested.

Heterogeneity was assessed by Q-statistics and *I* ^2^ statistics. While the Q-statistic analyses whether all studies share a common effect size, the *I* ^2^ value expresses the proportion of observed variance that is caused by variation in true effects rather than by sampling error.^(37)^ To investigate possible reasons for heterogeneity, a subgroup analysis was conducted according to the following criteria: short (≤ 56 days) *versus* long (> 56 days) study duration, low (< 200 mg/d) *versus* high (≥ 200 mg/d) dose of CoQ10, low (< 5) *versus* high (≥ 5) mean myopathy score at baseline, and having *versus* not having SAMS as inclusion criterion.

In addition to the subgroup analysis, a meta-regression was conducted to identify possible contributors to heterogeneity. Those are sample size, duration, dosage, and mean pain score at baseline. Meta-regression was conducted for intervention and control data separately. However, it should be stated that meta-regression with less than 10 studies is not recommended^(38)^ and results need to be interpreted with caution.

To assess publication bias, a funnel plot was created.^(37)^ Statistical analyses were carried out with the statistical software IBM SPSS version 29.0.1.0.^(171)^ Meta-regression was conducted with SPSS version 30.0.0.0.^(172)^

## Results

### Study characteristics

According to the eligibility criteria, seven randomised controlled double-blind intervention studies have been selected for inclusion in the review and meta-analysis. All selected trials investigate the impact of a daily oral CoQ10 supplementation on muscle pain under statin therapy.^(33,34,39–43)^ Six out of seven studies administer CoQ10 supplements in the intervention group and a placebo supplement in the control group.^(34,39–43)^ One trial compares the supplementation of CoQ10 in combination with n-3-fatty acids (n-3 FA) in the intervention group to a control group taking n-3 FA only.^(33)^ Data of 389 patients were analysed across all studies, with 202 patients in intervention groups and 187 patients in control groups. Both sexes were included in all studies, with a mean proportion of male patients of 50.25%. Patients of included studies had a mean age of 60.9 years.

Depending on the inclusion criteria of each RCT, different types of statins were administered (Simvastatin, Atorvastatin, Rosuvastatin, Lovastatin, Pravastatin, Fluvastatin), ranging from a dosage of 10 mg/d to 80 mg/d. The dosages of the CoQ10 supplementation ranged from 100 mg/d to 600 mg/d across all selected studies. Duration of the trials varied from 30 to 90 days.

Six studies have been conducted in outpatient clinics in Europe or the U.S.,^(33,34,40–43)^ while one study was performed in an European university setting.^(39)^ Further outcome criteria reported by the selected studies comprise the following measurements: CoQ10 serum concentration,^(39–42)^ mitochondrial markers (mitochondrial respiratory capacity, ROS production, citrate synthase activity) and CoQ10 muscle concentration,^(39)^ blood lipids, and CK,^(33,34,39–42)^ liver enzymes,^(34,40,42)^ and inflammatory markers.^(33)^ A detailed summary of the study characteristics of the seven RCTs is shown in Table 1.

**Table 1.** Characteristics of included randomised controlled trials

ID	Authors	Inclusion criteria	Exclusion criteria	Intervention/dose [mg/d]	Duration [d]	Control	Participants (i/c)	Age [mean ± SD] (i/c)	% male (i/c)	Statin type	Statin dose [mg/d]	Score
1	Dohlmann et al.^(39)^	Patients 40–70 yrs, BMI 25–35 kg/m^2^, simvastatin min. 40 mg/d in primary prevention	Disease history in serious medical disorders, mental disorders that interfere with the ability to understand, medication that impacts outcomes	CoQ10/400	56	Placebo	35 (18/17)	62.0 ± 1.0/64.0 ± 2.0	77.8/47.1	S	≥ 40	VAS
2	Derosa et al.^(40)^	Caucasian patients, ≥18 yrs, both sexes, not adequately controlled LDL-C levels + intolerance to statins	Serious medical conditions, weight change of >3 kg within last 3 mo, medication that impacts study outcome, pregnant/breastfeeding women	CoQ10/100	90	Placebo	60 (30/30)	59.8 ± 8.3/58.3 ± 7.9	43.3/50.0	S, A, R, L, P	10–40	VAS
3	Tóth et al.^(33)^	Patients with statin intake of at least 3 mo, with achievement of target LDL-C levels, increased level of TG	Adverse effects of statin intake that require dose reduction/discontinuation, intolerance of CoQ10/n-3 FA, disease history in secondary dyslipidaemia, conditions affecting prognosis/compliance of the protocol	CoQ10 + n-3 FA/200	90	n-3 FA	70 (35/35)	60.7 ± 12.4/ 60.7 ± 12.4	48.6/51.4	S, A, R, F	n/a	QBS
4	Taylor et al.^(41)^	Patients ≥20 yrs, both sexes, history of muscle complaints during statin intake	Disease history in serious medical conditions within last 5 yrs, medication that affects skeletal muscle metabolism	CoQ10/600	56	Placebo	38 (20/18)	58.0 ± 10.0/60.0 ± 10.0	n/a/ n/a	S	20	PSS
5	Skarlovnik et al.^(34)^	Patients 40–65 yrs, both sexes, statin-related muscle pain, use of statins for ≥ 6 mo + presence of muscular symptoms for ≥ 6 mo	Other causes for myopathy, disease history in hepatic/vascular/renal/endocrine disease, coagulopathy, current CoQ10 supplementation or anticoagulant therapy	CoQ10/100	30	Placebo	50 (25/25)	64.5 ± 9.5/65.6 ± 10.5	44.0/48.0	S, A, R, L, F	10–80	PSS
6	Fedacko et al.^(42)^	Statin-treated patients with muscle symptoms, with or without elevated levels of CK not leading to statin withdrawal	Hypersensitivity to study treatment, serious disease history + acute diseases, conditions that interfere with adherence to the protocol, CoQ10/selenium intake within last 3 mo	CoQ10/200	90	Placebo	60 (34/26)	59.6 ± 8.9/55.4 ± 12.4	35.3/26.9	S, A, R, F	n/a	VAS
7	Bookstaver et al.^(43)^	Patients on statin therapy, myalgia for ≥2 wks with no other causes, onset of pain within 60 d of initiation of the drug/dosage increase	Increased serum CK level, fibromyalgia, recent traumatic injury to the affected areas	CoQ10/120	90	Placebo	76 (40/36)	61.6 ± n/a/61.8 ± n/a	52.5/30.6	S, A, R, P	n/a	VAS

d, day/days; i, intervention; c, control; SD, standard deviation; yrs, years; BMI, body mass index; CoQ10, Coenzyme Q10; S, Simvastatin; VAS, Visual Analogue Scale; LDL-C, low density lipoprotein cholesterol; mo, months; A, Atorvastatin; L, Lovastatin; P, Pravastatin; R, Rosuvastatin; TG, triglycerides; n-3 FA, n-3 fatty acids; F, Fluvastatin; QBS, questionnaire by study team; n/a, not available; PSS, Pain Severity Score; CK, creatine kinase; wks, weeks.

### Qualitative evaluation

**Figure 2.**
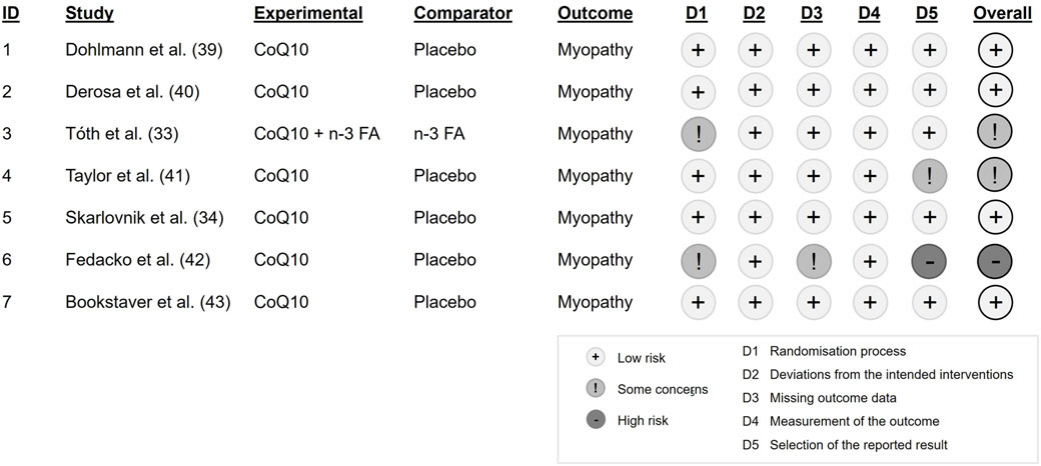
Risk of bias in individual studies.

**Figure 3.**
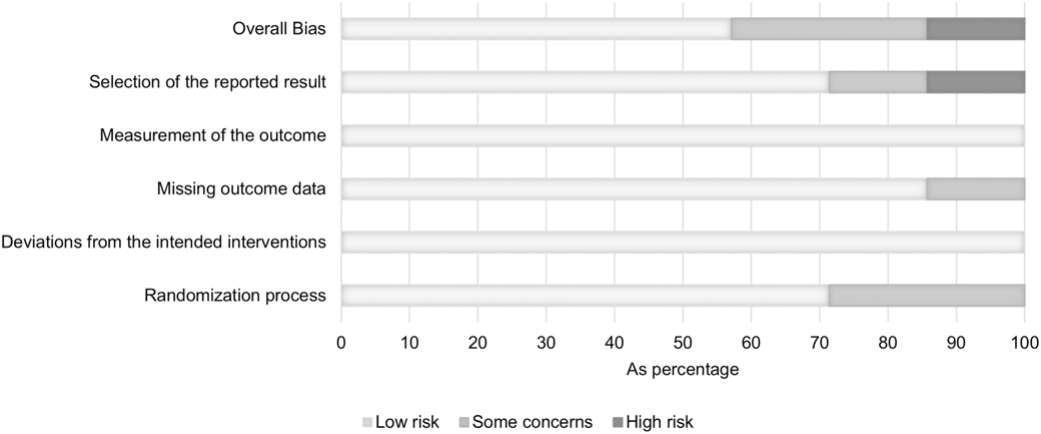
Risk of bias summary.

The risk of bias assessment shows an overall low risk of bias in four of the included studies with no considerable bias in all five underlying domains,^(34,39,40,43)^ whereas the remaining three studies show an overall moderate to high risk of bias (Figure 2).^(33,41,42)^ Some concerns result from the randomisation process in two studies, as the allocation of participants is claimed to be random but is not described in detail.^(33,42)^ Another concern arises from the domain “missing outcome data” in one study where data for the outcome of interest is only available for a subset of the participants.^(42)^ The domain “selection of reported results” causes a moderate to high risk of bias in two studies.^(41,42)^ In one study, a cross-over phase that was not part of the original protocol was added because less patients than expected experienced myalgia in a previous run-in-phase, resulting in a lower number of study participants than originally planned.^(41)^ The other study investigated the effects of both a CoQ10 and a selenium supplementation in double placebo-controlled subgroups but reports the results of the subgroups in a combined manner, not reporting the effects of the CoQ10 supplementation solely. Consequently, the overall risk of bias in this study is considered high.^(42)^ A summary of risk of bias is presented in Figure 3.

All included studies are single-centred trials. Of all seven studies, three studies reported a run-in period in which patients were asked to stop statin intake prior to the start of the trial to assure that the myopathic pain and other side effects are caused by the statin-intake.^(34,40,41)^ Four studies stated SAMS as inclusion criterion,^(34,41–43)^ while three studies included patients with and without SAMS besides other side effects.^(33,39,40)^ Among studies without SAMS as inclusion criterion, prevalences of myopathic symptoms were reported by Dohlmann et al.^(39)^ as follows: in the intervention group 11 out of 18 participants reported myalgia at baseline and in the control group 8 out of 17 participants.^(39)^ Derosa et al.^(40)^ reported that at baseline before wash-out and randomisation the prevalence of SAMS was 48 participants out of 60. In the study by Tóth et al.^(33)^ no information on SAMS prevalence in the study population was reported.

The intake of medication affecting the study outcomes, including pain medication, was considered in the eligibility criteria in four of the selected trials and led to exclusion of participants.^(34,39–41)^ While one trial did not exclude respective patients but reports the intake of pain medication as part of the study characteristics,^(43)^ two other trials do not report medication intake.^(33,42)^

### Meta-analysis

**Figure 4.**
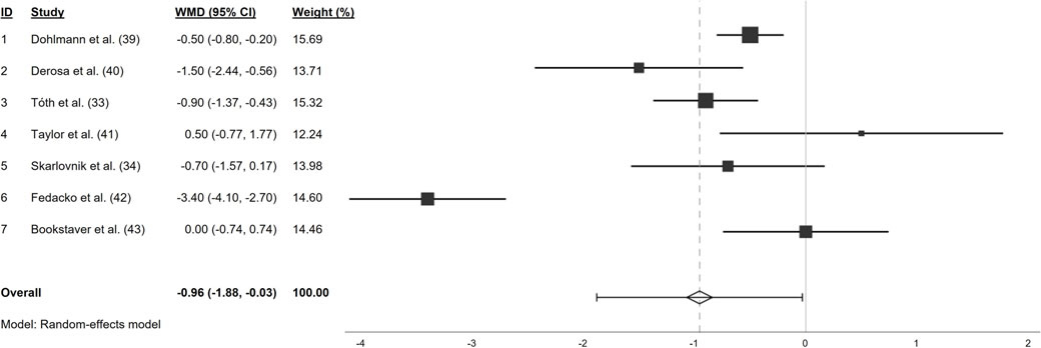
Forest plot of individual and pooled effects of Coenzyme Q10 compared to a control group on myopathic pain intensity (weighted mean difference, performed via random-effects model).

Four out of seven studies show a significant reduction of muscle pain intensity after CoQ10 supplementation compared to the control group,^(33,39,40,42)^ while three studies do not show a significant change of muscle pain intensity^(34,41,43)^ (Table 2). The overall effect size of this meta-analysis indicates a significant reduction of myopathic pain intensity by CoQ10 compared to a control group in a random effect model: WMD −0.96 (95 % CI −1.88; −0.03), *p* < 0.05 (Figure 4). A fixed effect model results in similar findings: WMD −0.84 (95 % CI −1.05; −0.63), *p* < 0.001. A leave-one-out-sensitivity analysis of the pooled effects is presented in Table 3. Repeating the meta-analysis with SDs that were calculated with different values for Corr (0.5 to 0.9) does not alter the results and the overall effect size remains significant (**Supplementary data 1**).

**Table 2.** Myopathic pain scores in individual studies

			Myopathic pain score						
			Pre-Intervention		Post-Intervention		Change		
ID	Study		Mean	SD	Mean	SD	Mean	SD	∆ Change (*p*-value)
1	Dohlmann et al.^(39)^	*i* *c*	2.4 1.4	0.7 0.5	2.2 1.7	0.6 0.6	− 0.2 + 0.3	0.5 0.4	− 0.5 (0.001)
2	Derosa et al.^(40)^	*i* *c*	5.3 5.6	2.4 2.7	3.1 4.9	1.2 2.2	− 2.2 − 0.7	1.8 1.9	− 1.5 (0.002)
3	Tóth et al.^(33)^	*i* *c*	3.9 3.9	1.2 1.2	2.7 3.6	0.7 1.6	− 1.2 − 0.3	0.9 1.1	− 0.9 (< 0.001)
4	Taylor et al.^(41)^	*i* *c*	0.0 0.0	0.0 0.0	2.2 1.7	2.3 2.4	+ 2.2 + 1.7	2.0 2.0	0.5 (0.44)
5	Skarlovnik et al.^(34)^	*i* *c*	3.9 3.5	2.0 2.4	2.9 3.2	2.0 2.1	− 1.0 − 0.3	1.3 1.8	− 0.7 (0.12)
6	Fedacko et al.^(42)^	*i* *c*	6.7 5.3	1.7 1.6	3.2 5.2	2.1 1.5	− 3.5 − 0.1	1.5 1.2	− 3.4 (< 0.001)
7	Bookstaver et al.^(43)^	*i* *c*	6.0 5.9	2.2 2.0	3.2 3.1	2.3 2.2	− 2.8 − 2.8	1.7 1.6	0.0 (1.00)

∆ change, difference of differences between groups as mean; i, intervention group; c, control group.

**Table 3.** Sensitivity analysis of overall intervention effects based on study selection (performed via random-effects model)

Study selection	WMD	95% Confidence Interval		*p*-value
		Lower	Upper	
All studies included	− 0.96	− 1.88	− 0.03	0.043
Without Dohlmann et al.^(39)^	− 1.03	− 2.13	0.06	0.063
Without Derosa et al.^(40)^	− 0.86	− 1.94	0.21	0.114
Without Tóth et al.^(33)^	− 0.96	− 2.07	0.15	0.089
Without Taylor et al.^(41)^	− 1.16	− 2.12	− 0.20	0.018
Without Skarlovnik et al.^(34)^	− 0.99	− 2.08	0.10	0.074
Without Fedacko et al.^(42)^	− 0.58	− 0.98	− 0.19	0.004
Without Bookstaver et al.^(43)^	− 1.12	− 2.14	− 0.09	0.033

WMD, weighted mean difference.

The analysis of heterogeneity discloses significant heterogeneity among the included studies, rejecting the hypothesis of a common effect size across all studies and showing a high variance due to true variance in effects (*Q* = 66.91; *I* ^2^; = 93.3 %, *p* < 0.001). The subgroup analysis reveals that both the study duration (short: WMD −0.47 (95 % CI −0.75; −0.19), *p* < 0.001 *versus* long: WMD −1.45 (95 % CI −2.86; −0.03), *p* < 0.05) and the dosage of CoQ10 (low: WMD −0.70 (95 % CI −1.55; 0.16), ns *versus* high: WMD −1.11 (95 % CI −2.70; 0.49), ns) do not have an impact on the overall results. The subgroup analysis for low (<5) versus high (>5) mean myopathy score at baseline revealed only a significant decrease in pain for the subgroup with low pain scores at baseline (WMD −0.59 (95 % CI −0.90; −0.28), *p* < 0.001). Though mean pain decrease was higher in the studies with higher mean baseline pain scores, this was not significant in the subgroup analysis (WMD −1.64 (95 % CI −3.59; 0.31), ns).

**Figure 5.**
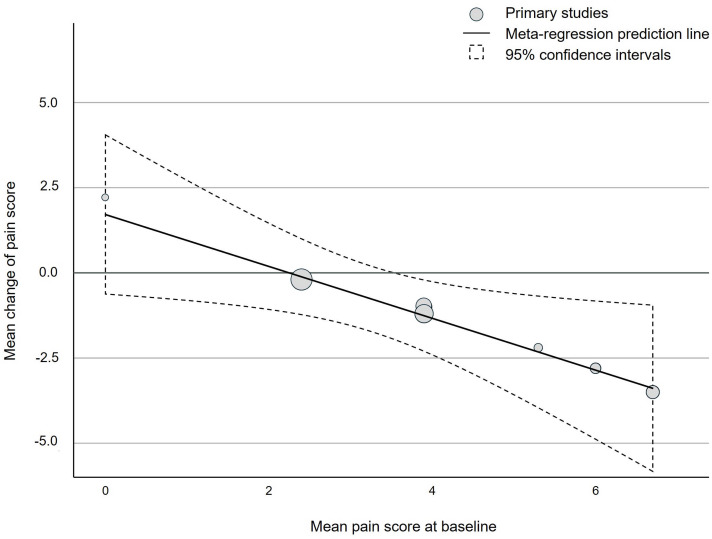
Bubble chart indicating a positive and significant association of mean baseline pain score and effectiveness of Coenzyme Q10 supplementation on pain reduction performed via meta-regression with intervention data only.

The results of the meta-regression indicate no significant associations between sample size (*p* = 0.69), study duration (*p* = 0.119), or CoQ10 dosage (*p* = 0.058) with mean change of pain score in the intervention groups, respectively. In contrast, data of intervention groups reveal a significant association between baseline pain score and intervention effect (*p* = 0.030) (Figure 5). For the control data no significant associations were found by meta-regression (data not shown).

Sub-group analysis comparing studies with and without SAMS as inclusion criterion show a non-significant reduction in mean pain score (WMD −0.935 (95 % CI −2.654; 0.785), ns) for studies with SAMS as inclusion criterion, while studies without SAMS as specific inclusion criterion show a significant pain reduction (WMD −0.823 (95 % CI −1.302; −0.345), *p* < 0.001).

**Figure 6.**
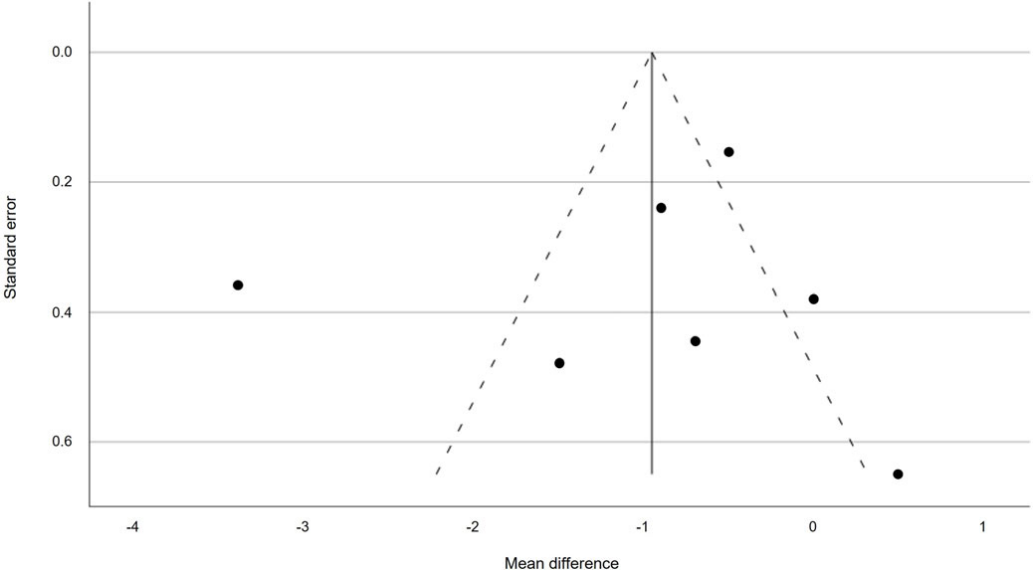
Funnel plot of potential publication bias.

The existence of publication bias was assessed via a funnel plot and the outcome is presented in Figure 6.

## Discussion

The results of the present meta-analysis reveal an overall significant reduction in muscle pain intensity after CoQ10 supplementation compared to a control treatment in patients with existing statin-induced myopathy. Previous meta-analyses have shown contradicting results, with one meta-analysis demonstrating a beneficial effect^(26)^ and three meta-analyses indicating no effect of a CoQ10 supplementation on SAMS.^(24,25,27)^ The present meta-analysis focuses on seven RCTs including the most recent publications, thus bringing the debate on the effect of a CoQ10 supplementation up to date. Despite a positive intervention effect, a high heterogeneity was detected. Thus, a deviation from a common effect size and the influence of additional factors is most likely.^(37)^ The subgroup and meta-regression analyses that were conducted to investigate possible influencing factors do not provide evidence for the study duration, sample size and CoQ10 dosage. Meta-regression and subgroup analysis reveal a possible impact of baseline pain scores on the effect of the supplementation. Further studies should focus on patients having moderate to severe pain scores at baseline that are clearly associated with statin intake. Further, intake of pain medication should be an important outcome measure. However, the evidence of the subgroup analysis and meta-regression is limited due to the small number of studies.^(37,38)^

Beside the aspects that have been investigated in the subgroup analysis, further factors such as the type and dose of statins may have an impact on the intervention effect. Previous investigations show that the risk of developing myopathy is dose-dependent, and statin-type is a predictor for SAMS, with higher numbers of SAMS in patients administered to lipophilic statins.^(10,44)^ A subgroup analysis that includes both statin-dose and -type was, however, not possible as the RCTs merge different types and doses of statins. Further, it would be interesting to examine the intervention effect of CoQ10 formulations differing in their redox status (ubiquinone as oxidised form, ubiquinol as reduced form).^(45)^ Since this information was not given in the included studies, a potential effect of CoQ10 formulation could not be investigated in a subgroup analysis either. Consequently, intervention effects depending on statin type, statin dose and CoQ10 formulations could not be evaluated comprehensively.

Variations in the study designs of the selected RCTs may further confound intervention effects.^(38)^ All studies are single-centred, which limits the broader transferability of the results. In addition, all trials have a small sample size and do not exceed a duration of 90 days. Thus, effects of supplementation might not have been detectable in all studies. Further, an exclusion of patients without statin-induced pain was performed only in four studies^(34,41–43)^ and among those only one confirmed SAMS by having a wash-out period.^(41)^ Because muscle pain is often nonspecific and may occur in association with other conditions,^(11)^ the presence of true statin-induced myopathy was not guaranteed in the remaining studies.^(34,42,43)^ In addition, the use of other medications or analgesics may have influenced the perception of muscle pain.^(11)^ As medication intake was only investigated in four studies,^(34,39–41)^ effects of the intervention on the intensity of muscle pain might be underestimated in the remaining trials.^(33,42,43)^ The fact that some studies included patients with and without SAMS^(33,39,40)^ could further have contributed to underestimation of the effectiveness of CoQ10 supplementation in this meta-analysis. Still the effect of the supplementation was highly significant in this subgroup.

Despite strict inclusion criteria, additional supplementation with n-3 FA and selenium in the studies of Tóth et al.^(33)^ and Fedacko et al.,^(42)^ respectively, may have influenced the results.^(33,42)^ Tóth et al.^(33)^ hypothesise an additive effect of joint CoQ10 and n-3 FA administration.^(33)^ In the trial of Fedacko et al.,^(42)^ selenium as CoQ10 restoring and antioxidant agent is supplemented in addition to CoQ10. This study showed a strong beneficial effect in the analysis.^(42)^ Contradictory results were found in the study by Taylor et al.,^(41)^ in which patients underwent an extensive lead-in phase and did not experience muscle pain at intervention start. In this study, statin-intake caused an increased muscle pain intensity in the course of the trial, which was higher in the intervention group.^(41)^

The implemented studies used different tools for pain assessment. Four studies used VAS, two studies used PSS, and one study used an own questionnaire. However, results of the scores are comparable as all scores assess pain only, namely with values from 0 to 10. For further studies, VAS and PSS should be used for better comparison with other studies.

To examine the overarching influence of each individual trial, a leave-one-out sensitivity analysis was performed. The results show that the overall effect size of the meta-analysis varies with study selection. Despite of some limitations, results indicate beneficial effects of CoQ10 supplementation in patients with statin-induced muscle pain, which is in accordance with additional clinical and biochemical data.^(46,47)^

A meta-analysis by Banach et al.^(48)^ shows an overall reduction of serum CoQ10 levels by − 0.44 µmol/L (95% CI − 0.52; −0.37, *p* < 0.001) caused by statins.^(48)^ However, investigations of muscle biopsies showed that intramuscular CoQ10 levels are decreased in some, but not all statin-treated patients.^(49–51)^ In the study by Dohlmann et al.^(39)^ a CoQ10 supplementation could not increase intramuscular CoQ10 levels in statin-treated patients.^(39)^ It remains unclear to what extend an oral supplementation influences intramuscular CoQ10 levels. Supplements with a good bioavailability at adequately high doses are required to improve tissue CoQ10 levels and to ensure intervention effectiveness.^(45)^ In addition to intramuscular CoQ10 levels, Dohlmann et al.^(39)^ focused on the effects of CoQ10 supplementation on mitochondrial function in muscle cells. The authors could not find effects on citrate synthase activity, ROS production, or mitochondrial capacity for oxidative phosphorylation after CoQ10 supplementation compared to placebo. However, the results need to be verified in trials with larger sample sizes.^(39)^ Further studies indicate beneficial effects of CoQ10 supplementation on other mitochondrial markers such as the L/P ratio, with a significant reduction in one study,^(46)^ a non-significant trend of reduction in two studies,^(52,53)^ and with an increase of L/P ratio in the control group but not in the intervention group in another trial.^(54)^ However, among these studies, myopathy is an inclusion criterion only in one study^(53)^ and not all patients in another study take statins.^(46)^ In future studies CoQ10 levels should be measured. It is most likely that persons with low CoQ10 levels benefit the most from a CoQ10 supplementation. Additionally, compliance of taking CoQ10 supplements as well as statin medication might have an impact on study results and should be considered in future studies.

Trials that investigate the pain interference with daily life activities show a significant improvement after the intervention.^(34,47)^ Consequently, a CoQ10 supplementation can contribute to patient’s well-being, which is a substantial factor for treatment continuation and cardiovascular risk reduction.^(40)^ Since CoQ10 has multifaceted properties, CoQ10 has been discussed for the prevention and treatment of various chronic and age-associated diseases.^(55)^ Older age is associated with lower CoQ10 levels, which are further reduced by comorbidities and drug intake.^(56,57)^ Animal-based foods are the main nutritional source of CoQ10. Intake of CoQ10 from food ranges from 3–6 mg/d, which is not sufficient to cover increased requirements.^(58)^ A CoQ10 supplementation is generally considered safe with no remarkable side effects even up to high doses of 1200 mg/d, thus a supplementation of CoQ10 could be used to prevent a CoQ10 deficiency especially additionally to statin treatment.^(59)^ In regard to a continuously aging population, CoQ10 supplementation could possibly offer a cost-effective and low-threshold option to assure adequate CoQ10 levels and to support the treatment of chronic metabolic and degenerative diseases.^(57,60)^

## Conclusion

This meta-analysis shows a significant effect of CoQ10 supplementation compared to placebo on reducing myopathic pain intensity in statin-treated patients with SAMS. However, the results are limited due to small sample sizes and a high heterogeneity. Thus, more well designed studies with a larger sample size and participants with higher pain scores that are clearly associated with statin intake are necessary, most preferable with a multi-centre study design. Intake of pain medication, CoQ10 levels at baseline, and compliance to the study protocol should be considered as additional outcome criteria or confounders in future studies. Nevertheless, the outcome of this meta-analysis highlights the potential of a CoQ10 supplementation as a safe and cost-effective option to minimise adverse effects of statin intake and to improve patients’ quality of life.

## Supporting information

Kovacic et al. supplementary material Kovacic et al. supplementary material

## Supplementary material

To view supplementary material for this article, please visit https://doi.org/10.1017/jns.2025.10043
