# Supplementary material for: Effects of coenzyme Q10 supplementation on myopathy in statin-treated patients: a systematic review and meta-analysis
Source: J Nutr Sci. 2025 Oct 10;14:e72. doi: 10.1017/jns.2025.10043 (PMC12554813; doi:10.1017/jns.2025.10043)
Supplement: Kovacic et al. supplementary material [file S2048679025100438sup001.docx]

## Supplementary data 1: Search terms – statins and Coenzyme Q10

**Table S 1** Search terms (free-hand and MeSH-terms) for literature research of statins combined with Coenzyme Q10

| Search run | Search terms |
| --- | --- |
| total | ((Statin*) OR ("HMG-CoA reductase inhibitor") OR ("Cholesterol synthesis inhibitor")) AND (("Coenzyme Q10") OR (Ubiquino*))  ("Hydroxymethylglutaryl-CoA Reductase Inhibitors"[Mesh]) AND (("coenzyme Q10" [Supplementary Concept]) OR ("Ubiquinone"[Mesh])) |
| with myopathy | ((Statin*) OR ("HMG-CoA reductase inhibitor") OR ("Cholesterol synthesis inhibitor")) AND (("Coenzyme Q10") OR (Ubiquino*)) AND ((Myopath*) OR (Myalg*))  ("Hydroxymethylglutaryl-CoA Reductase Inhibitors"[Mesh]) AND (("coenzyme Q10" [Supplementary Concept]) OR ("Ubiquinone"[Mesh])) AND ("Muscular Diseases"[Mesh]) |
| without myopathy | ((Statin*) OR ("HMG-CoA reductase inhibitor") OR ("Cholesterol synthesis inhibitor")) AND (("Coenzyme Q10") OR (Ubiquino*)) NOT ((Myopath*) OR (Myalg*))  ("Hydroxymethylglutaryl-CoA Reductase Inhibitors"[Mesh]) AND (("coenzyme Q10" [Supplementary Concept]) OR ("Ubiquinone"[Mesh])) NOT ("Muscular Diseases"[Mesh]) |

*MeSH, Medical Subject Headings*

## Supplementary data 2: Meta-analysis – different correlation coefficients

**Table S 2** Meta-analysis results based on calculations with different correlation coefficients

| Corr | WMD | 95% Confidence Interval | | | p-value | I² [%] |
| --- | --- | --- | --- | --- | --- | --- |
|  |  | lower | upper | |  |  |
| 0.5 | -0.95 | -1.87 | | -0.02 | 0.045 | 88.8 |
| 0.6 | -0.95 | -1.87 | | -0.03 | 0.044 | 91.1 |
| 0.8 | -0.96 | -1.89 | | -0.04 | 0.042 | 95.0 |
| 0.9 | -0.97 | -1.89 | | -0.04 | 0.041 | 97.2 |

*Corr, correlation coefficient; WMD, mean difference*
